# TNFSF14: LIGHTing the Way for Effective Cancer Immunotherapy

**DOI:** 10.3389/fimmu.2020.00922

**Published:** 2020-05-15

**Authors:** Joseph G. Skeate, Mikk E. Otsmaa, Ruben Prins, Daniel J. Fernandez, Diane M. Da Silva, W. Martin Kast

**Affiliations:** ^1^Department of Molecular Microbiology and Immunology, Keck School of Medicine, University of Southern California, Los Angeles, CA, United States; ^2^Department of Obstetrics and Gynecology, Keck School of Medicine, University of Southern California, Los Angeles, CA, United States; ^3^Norris Comprehensive Cancer Center, University of Southern California, Los Angeles, CA, United States

**Keywords:** tumor necrosis factor superfamily member 14 (TNFSF14), LIGHT, CD258, cancer immunotherapy, tumor microenvironment

## Abstract

Tumor necrosis factor superfamily member 14 (LIGHT) has been in pre-clinical development for over a decade and shows promise as a modality of enhancing treatment approaches in the field of cancer immunotherapy. To date, LIGHT has been used to combat cancer in multiple tumor models where it can be combined with other immunotherapy modalities to clear established solid tumors as well as treat metastatic events. When LIGHT molecules are delivered to or expressed within tumors they cause significant changes in the tumor microenvironment that are primarily driven through vascular normalization and generation of tertiary lymphoid structures. These changes can synergize with methods that induce or support anti-tumor immune responses, such as checkpoint inhibitors and/or tumor vaccines, to greatly improve immunotherapeutic strategies against cancer. While investigators have utilized multiple vectors to LIGHT-up tumor tissues, there are still improvements needed and components to be found within a human tumor microenvironment that may impede translational efforts. This review addresses the current state of this field.

## Introduction

Cancer remains as one of the most significant medical challenges for human beings and accounts for 1 out of every 6 deaths (1). In the United States it is estimated that 39% of people will develop cancer, and given the aging population we can assume that the cancer incidence rate will remain a significant burden for humankind (2). As such, the need for new therapies that target cancer remains at the epicenter of medical research. Compared to current standards of care such as chemotherapy, surgery, and radiation; immunotherapies have brought to the table a new set of tools and strategies that have expanded the scope of cancer treatment options. The main goals of cancer immunotherapy can be broken down into three separate approaches: generation of *de novo* anti-cancer immune responses, bolstering/amplification of ongoing immune responses, and the prevention of cancers from shutting down/manipulating anti-tumor responses. While there has been significant progress made in our understanding of how tumors evade immune-based interventions, the generation of specific anti-tumor responses alone remains to be insufficient to clear solid tumors as T cells often fail to traffic to and infiltrate tumor sites. These shortcomings are compounded by the immunosuppressive nature of the tumor microenvironment itself and by associated immune suppressor cells, which makes it difficult for even checkpoint inhibitor-based therapies to be entirely effective. This review addresses how Tumor Necrosis Factor Superfamily member 14 (TNFSF14/CD258), otherwise known as LIGHT, could potentially be used to counteract these aforementioned shortcomings.

Intratumoral LIGHT expression is highly effective in driving anti-tumor immune responses while also eliciting significant changes to the tumor microenvironment. In this review, we will summarize the known effects that LIGHT has on tumor immunobiology and highlight the findings, expression vectors strategies, and immunotherapy combinations researchers have used over the years to “LIGHT-up” the tumor microenvironment as well as provide considerations that should be taken into account for future LIGHT-based vector designs.

### LIGHT

LIGHT (homologous to **l**ymphotoxin, exhibits **i**nducible expression and competes with Herpes Simplex Virus **g**lycoprotein D for **H**erpes Virus Entry Mediator, a receptor expressed by **T** cells), is a protein primarily expressed on activated T cells, activated Natural Killer (NK) cells, and immature dendritic cells (DC) (3, 4). Approximately 29 kD in size, LIGHT can function as both a soluble and cell surface-bound type II membrane protein and must be in its homotrimeric form to interact with its two primary functional receptors: Herpes Virus Entry Mediator (HVEM) and Lymphotoxin-β Receptor (LTβR) (3, 5, 6). LIGHT signaling through these receptors have distinct functions that are cell-type dependent, but interactions with both types of receptors have immune-related implications in tumor biology.

LIGHT-HVEM interaction is responsible for a majority of the immune-stimulating properties of LIGHT (7). Expressed on lymphocytes, NK cells, smooth muscle, and epithelium, HVEM serves as an important T cell costimulatory agent leading to activation, proliferation, and survival (4, 8, 9). HVEM can also trigger NK cells to produce IFNγ through LIGHT-mediated nuclear factor-κB (NFκB) RelA/p50 signaling (7, 8, 10, 11). Furthermore, LIGHT produced by tumor-sensing NK cells is a critical component in the NK-DC crosstalk that occurs in the priming of *de novo* anti-tumor responses (12). To activate T effector cells, HVEM is necessary for LIGHT's costimulatory effect in a CD28-independent T cell to T cell manner (4). Such pro-inflammatory HVEM interactions increase the expression of Th1 cytokines IFNγ and GM-CSF. As such, LIGHT-HVEM mediated T cell co-stimulation and NK-DC crosstalk both play a vital role in generating anti-tumor immunity in a therapeutic context (13).

The other receptor, LTβR, is found on the surface of epithelial, stromal, immature DC, and myeloid cells, but not on lymphocytes (14). During normal biological development LIGHT-LTβR interactions have been identified as a component of lymphoid structure development and maintenance (15). In the context of anti-tumor immune support, LIGHT-LTβR signaling has a wide range of roles that span from influencing cancer cells' susceptibility to immune responses, functioning to repair chaotic tumor vasculature, and to supporting effector cells cell trafficking to and infiltration into tumors. If we consider LIGHT-HVEM the primary driver of anti-tumor immune activity, then LIGHT-LTβR functions to build-out, repair, and maintain the infrastructure needed to support these immune responses.

## Effects of LIGHT on Tumor Biology

**Figure 1:**
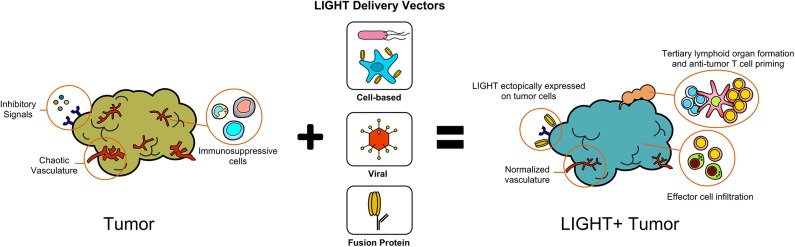
Delivery of LIGHT through different methods modifies the tumor microenvironment.

The expression of LIGHT within tumors has profound effects on host immune responses against tumors and remodeling of the TME (Figure 1). In addition to sensitizing tumor cells to IFNγ-mediated apoptosis, LIGHT induces tumor vasculature normalization, and drives the formation of high endothelial venules which subsequently encourage generation of tertiary lymphoid structures (TLS) (16–18). In addition, LIGHT stimulates effector cell function and antitumor CD8^+^ T cell entry into tumors, which aids in establishing anti-tumoral memory (19–22). In this section, we will summarize the critical roles that LIGHT can play in remodeling tumor architecture while also driving anti-tumor immunity.

### Tumor Vascular Normalization Occurs With Targeted LIGHT Treatments

Healthy vasculature allows constant blood flow, oxygen perfusion, and circulation of immune cells; features which tumor vasculature lacks (23). As tumor cells divide, hypoxic pockets develop within the tumor mass. Tumor cells within these hypoxic zones respond by overexpressing pro-angiogenic factors such as members of the vascular endothelial growth factor (VEGF) family to modify nearby stromal cells (endothelial cells, pericytes, vascular smooth muscle cells, fibroblasts) (24, 25). Through this mechanism tumors accommodate their increasing metabolic requirements by extending existing healthy blood vessels through angiogenesis, however tumor cells can also undergo trans-differentiation into an endothelial-like phenotype. They use this phenotypic switching mechanism to create a blood circulation network through a process known as vascular mimicry (26). Furthermore, production of VEGF-protein family members downregulates effector lymphocyte attachment molecules such as intracellular adhesion molecules (ICAMs) and vascular cell adhesion molecules (VCAMs), supporting direct elimination of effector cells by T regulatory (Treg) cells through FAS/FASL interactions due to changes in the ratio of effector to suppressor cells, a problem that is further exacerbated by the tumor recruiting suppressive cells through the release of molecules such as CCL28 and CCL2 (Treg and myeloid derived suppressor cell chemo-attractants) (27–29). The combined effect of this less perfuse, transfigured vascular basement membrane, and enhanced level of suppressive cell recruitment creates a significant barrier that prevents effector cell infiltration and function (24).

When the vasculature within a tumor is normalized toward a non-pathogenic phenotype, it has been shown to alleviate hypoxia, intra-tumoral pressure, and improves almost all treatment options whether they are immunotherapy, radiotherapy, or chemotherapies (30). LIGHT-based therapies developed by Johansson-Percival et al. were found to combat tumor vasculature not by destroying tumor stroma, but by reversing their pathogenic effects through vascular normalization (21–23). Although the exact mechanisms remain unclear, evidence has shown LIGHT, when delivered as a fusion protein linked to a tumor vascular targeting peptide (VTP), can normalize intra-tumoral blood vessels via increased expression of the LTβR dependent pericyte contractile markers ICAM-1, VCAM-1, smooth muscle actin (SMA), calponin, and caldesmon (21–23). Such contractile markers make tighter cellular junctions, thus creating a less “leaky” phenotype. The intra-tumoral macrophages activated by LIGHT were found to secrete TGF-β, which induced a vascular smooth muscle cell (vSMC) phenotype switch and increased adhesion maker expression in a Rho-kinase dependent manner (21). TGF-β is also responsible for the differentiation of pericytes, explaining the increased pericyte contractile markers found in LIGHT treated tumors (8, 31, 32). The researchers hypothesized that the secreted TGF-β was unable to cause pro-tumor effects because macrophage-secreted TGF-β is released so closely to stromal cells that it is unable to diffuse throughout the tumor. Overall, this LIGHT-driven vascular normalization has been shown to improve pericyte and/or vSMC markers in murine pancreatic insulinoma, breast cancer, glioblastoma, melanoma, Lewis Lung carcinoma (LLC), and metastatic B16 melanoma models, in addition to human glioblastoma and astrocytoma models, rendering them more susceptible to cancer treatments (21–23, 33).

### Presence of LIGHT Gives Rise to a More Inflamed Tumor Microenvironment

The tumor microenvironment (TME) is the result of biological crosstalk between stromal, cancer, and immune cells within a given tissue (34, 35). Based on the heterogeneity that tumors develop, they take on a sub classification of being either “hot” or “cold,” which is ultimately dictated by the ability of the immune system to recognize, infiltrate, and function against their growth. The inability to recognize cold tumors arises from a set of compounding factors in the TME: lack of response to tumor antigens, homing, maturation, and function of antigen presenting cells, or failure of effector responses to infiltrate or function against tumors due to immunosuppressive cell populations [reviewed in Bonaventura et al. (36)]. Immunologically cold tumors are populated with a myriad of immune suppressor cells such as tumor-associated macrophages (TAM), Tregs, and myeloid derived suppressor cells (MDSC). Each of these populations can impair effector cell generation or function through either direct interaction, the production of immunosuppressive cytokines (e.g., TGF-β and IL-10), or a combination of the two (37–39). Additionally, the tumor itself may influence effector cell function through the expression of signals such as PD-L1 in response to exposure of elevated IFNγ levels (39, 40).

As a hallmark of successful LIGHT therapy designs, researchers have repeatedly shown a LIGHT-dependent increase in intratumoral IFNγ, TNFα, MIG, and IP-10, all of which are indicative of effector cell responses and are cytokines that profile tumors as “hot” (10, 19, 20). This direct change of the tumors immunological phenotype is driven by the effects LIGHT exerts on the TME. First, the normalizing of the tumor vasculature through LIGHT-LTβR signaling described in the last section allows for decreased levels of tumor hypoxia and intra-tumoral pressure. This directly limits the tumors ability to recruit and generate immune suppressor cells within the TME while at the same time encouraging effector cell recruitment and ability to function. Second, LIGHT-LTβR signaling is responsible for creating High Endothelial Venules (HEVs), the primary sites for leukocyte extravasation into target tissues (15). Cells that make up LIGHT-driven HEV structures express mucosal vascular addressin cell adhesion molecule 1 (MAdCAM1) as well as peripheral node addressins (PNAd), which bind L-selectin on lymphocytes and facilitate effector cell entry (22). Additionally, the production of CCL21 by the HEV endothelial cells recruits naïve CCR7+ T cells to tumor sites, which are essential in the generation of anti-tumor immunity (15, 41). Given that the presence of tumor infiltrating lymphocytes (TIL) have been posited with better outcomes in cancer models such as melanoma, breast, ovarian, colorectal, and lung (42, 43), LIGHT-LTβR induced construction of HEVs are clinically relevant. Staining for MECA 79 expression (a PNAd marker) to reveal *de novo* generation of these structures has occurred in pancreatic, breast, and glioblastoma models that have undergone LIGHT-based treatments (24). This increased lymphoid penetration also leads to other structural changes in the tumor microenvironment, such as the development of TLSs (16).

Johansson-Percival et al. demonstrated that one of the indicators of successful anti-tumor immunity in LIGHT therapy was the formation of TLSs within a rat insulin promoter (RIP)1-Tag5 pancreatic insulinoma mouse model (22, 44). TLS (sometimes referred to as tertiary lymphoid organs), are a subset of lymphoid tissues that arise in sites of chronic inflammation and have been associated with autoimmune diseases (45). TLS are similar to secondary lymphoid organs (SLO), such as lymph nodes, as they are made up of compartmentalized T and B cell germinal centers. But unlike SLOs, TLSs are not encapsulated and lack afferent lymph vessels, allowing them to directly interact with external antigens within the immediate environment (8, 45). TLS are formed in association with the overexpression of lymphocyte and DC chemokines CCL21 and CCL19 as well as HEV markers MAdCAM1 and PNAd: all of which are dependent on LTβR signaling (15, 45, 46). Once formed, TLS within or around tumors function as sites for processing tumor antigens, which are released by dying tumor cells or those that are killed by NK cells activated through LIGHT-HVEM interactions (8, 46). Presentation of these tumor antigens by activated DC then results in the generation and expansion of tumor-specific CD8^+^ effector cells, the population of cells responsible for LIGHT-driven tumor regression. Importantly, mice that received LIGHT-based therapy rejected distal tumors and were resistant to re-challenges after primary tumor clearance, highlighting the existence of memory responses (10, 19, 20, 47, 48). It is worth noting that outside of LIGHT-based therapies the *de novo* generation of TLS in murine tumor models has been limited [reviewed in (49, 50)]. Importantly, however, the presence of TLS has been associated with positive clinical outcomes in a large number of human cancers and has can serve as a biomarker for successful immunotherapeutic approaches (51, 52).

Taken together, LIGHT-mediated correction of tumor vasculature along with generation of sites for lymphocyte entry and effector cell expansion can work together to shift a cold TME to one that is immunologically hot and may be susceptible to proper therapy interventions. In the next section we review the approaches that investigators have taken to deliver LIGHT to tumor sites as well highlight successful combination approaches.

## Light Delivery Systems

Over the past two decades, researchers have investigated the use of gene transduction, adoptive transfers, viral vectors, and peptides as delivery systems for LIGHT therapies. Through the development and utilization of these vectors, researchers have been able to piece together how LIGHT mediates its anti-tumor effects and the extent to which it may be combined with other treatment options to overcome challenging tumor models. The details, including the vector, tumor models tested, delivery route, results, and whether the vectors were used in combination with another modality of treatment are summarized in Table 1.

**Table 1:** Systems used to deliver LIGHT to tumors with tumor models, delivery routes, combinations, and summary outcomes.

**Platform**	**Construct**	**Cancer models**	**Administration route**	**Combinations**	**Effects on tumor**	**References**
Bacterial	*Salmonella typhimurium* expressing LIGHT	D2F2 breast carcinoma	IV	-	Reduced tumor volume	(53)
		CT-26	IV	-	Reduced tumor volume	(53)
		Lewis lung carcinoma	IV	-	Reduced tumor volume	(53)
Viral	Adenovirus delivery of LIGHT (Ad-LIGHT)	Ag104Ld	Intratumoral injection (IT)	-	Primary tumor elimination and distal tumor clearance	(20)
		4T1	IT	-	Primary tumor clearance and elimination of metastatic events	(20)
		MC38	IT	-	Primary tumor elimination	(20)
		B16-SIY	IT	-	Primary tumor elimination	(20)
		A20	IT	-	Primary tumor clearance and protection from rechallenge	(54)
		C3.43 HPV16 cervical cancer	IT	Tumor Vaccine (VRP w/HPV16E7)	Tumor size regression, combination showed enhanced efficacy. Therapeutic treatment provided protection from rechallenge	(19)
		TRAMPC2 prostate cancer	IT	Tumor Peptide vaccine	Tumor size regression, LIGHT reduced effect of Tregs. Enhanced anti-tumor effects with combination treatments	(55)
Cells	Mesenchymal stem cells expressing LIGHT	TUBO mammary cancer	IV	-	LTbR and CD8-dependent prophylactic protection against tumor challenge as well as therapeutic efficacy against day-7 tumor growth	(56)
Fusion Protein	LIGHT linked to a vascular targeting peptide (LIGHT-VTP)	Pancreatic insulinoma (RIP1-Tag5)	IV	Tumor vaccine (Tag-CpG-ODN) + Anti-PD-1 & CTLA-4	Significant reduction in tumor burden of mice receiving full combination treatments. LIGHT-therapies enhanced tumor vaccine + dual checkpoint blockade	(22)
		Lewis lung Carcinoma	IV	Anti-PD-1 & CTLA-4	Reduced tumor burden in mice receiving triple therapy compared to controls. No necrosis in tumors indicating improved vasculature	(22)
		NFpp10-Glioblastoma multiforme (GBM)	IV	Anti-VEGF + Anti-PD-1	HEV formation, vasculature normalization, enhanced levels of CD3+ cell infiltration into tumors, upregulations of granzyme B, and reduction in Tregs	(23)
		B16 melanoma	IV	Anti-PD-1	Vascular normalization in both primary and lung metastases. Reduced number of metastases accompanied by TLO and HEV formation at metastatic sites. Sensitization to anti-PD-1 treatments	(33)
Fusion Protein	Three copies of LIGHT linked to scFv targeting EGFR (anti-EGFR-hmLIGHT)	Ag104Ld	IV	Anti-PD-L1	Significant reduction in tumor size within combination group showing the ability to overcome checkpoint-blockade resistance	(57)
		MC38	IV	Anti-PD-L1	Tumor clearance with combination therapy	(57)

### Gene Transduction to Create LIGHT-Expressing Tumors

Researchers first assessed LIGHT's *in vivo* abilities to reduce cancer burden via direct transfection of tumor cells and adoptively transferring them into mice. Ag104Ld is an aggressive fibrosarcoma that is unaffected by most immunotherapies, and has been a popular model for testing the effects of LIGHT (57). Papers by Yu et al. and Fan et al. demonstrated that Ag104Ld tumors expressing LIGHT are rejected in an immunocompetent setting and mice become resistant to re-challenge with the parental Ag104Ld cell line at 8-weeks post initial tumor clearance (10, 20, 48). Intratumoral anti-tumor T cell priming and expansion, most likely due to TLS formation, was seen by Yu et al. through the usage of a T cell receptor (TCR)-transgenic cell line, 2C, that can only be activated by interaction with the Ld antigen directly on Ag104Ld tumors. In a primary Ag104Ld or Ag104Ld LIGHT^+^ tumor challenge followed by a distal Ag104Ld challenge, Yu et al. found up to 100x more intra-tumoral 2C T cells in distal metastasis sites of Ag104Ld LIGHT^+^ mice than the control (20). This influx of 2C T cells in distal tumor sites demonstrated direct Ag104Ld T cell priming via LIGHT stimulation within primary tumors.

Fan et al. established an additional layer in the priming process that highlights the vital role of LIGHT-HVEM interaction in the Ag104Ld LIGHT^+^ model. They found that LIGHT activates NK cells through the HVEM receptor, leading to the activation of CD8^+^ cells in an IFNγ-dependent manner (10). Furthermore, Zhai et al. forced LIGHT expression in MDA-MB-231 human breast carcinoma cells via a retroviral vector and found significant inhibition of tumor growth when compared to controls (58). Qiao et al. transfected CT26 colorectal cancer models to express LIGHT constitutively, resulting in a stunted tumor growth, lower distal liver metastasis burden, and prevention of tumor take in re-challenge events (47). Further investigation showed a marked increase in tumor infiltrating lymphocytes, increased IFNγ levels, and higher concentrations of the DC activation marker CD86 in LIGHT-expressing tumors when compared to control (47). With the literature establishing that the expression of LIGHT by tumors leads to a CD8-dependent clearance of the primary tumor and generates long-lasting memory against LIGHT-negative parental cell lines, additional methods were sought to specifically deliver LIGHT to tumor sites or force express LIGHT in tumors.

### Adenovirus Vectors

The use of replication-deficient viruses, such as the adeno-associated virus, have been used to generate potent immunogenic responses with minimal toxicity (59, 60). Given their promiscuity in cell binding, as well as their ability to force cellular expression of target proteins, they represent viable vectors for the forced expression of proteins of interest within targeted sites (8). Following *in vitro* success of adenoviruses carrying LIGHT (Ad-LIGHT) to inhibit tumor growth, researchers have been able to elicit robust anti-tumor responses *in vivo* (61). In 2007, Yu et al. showed rejection of established tumors as well as distal metastases with an intra-tumoral adenovirus injection that resulted in the expression of LIGHT (Ad-LIGHT) (20). The tumor models that have been successfully treated through this modality include the aggressive fibrosarcoma Ag104Ld and mammary carcinoma 4T1 cell lines. Within the tumors, researchers found increased tumor specific CD8^+^ T cell infiltration and high levels of IFNγ and TNFα when compared to an adenovirus control and no treatment. Our group has specifically shown successful therapy through adenovirus delivery of LIGHT within the HPV-transformed cervical cancer model C3.43 as well as in the TRAMP-C2 prostate cancer model (19, 55). While this vector was able to show gene-transduction of LIGHT and subsequent anti-tumor responses, it relies on direct injection of the vector into primary tumor sites and lacks the ability to be delivered systemically due to target cell binding promiscuity.

### Cell-Based Vectors for LIGHT

Adoptive cell transfer methods offer a unique approach to delivering a payload to tumor sites. One such method of LIGHT-delivery that has been investigated took advantage of the tumor targeting properties of *Salmonella*. Specific strains of this bacterium have been shown to colonize and grow within tumors; most likely due to the tumors' hypoxic nature. Low oxygen regions within the TME can nurture the growth of facultative anaerobes and, given the ease in which genetic material of *Salmonella* can be manipulated, this vector has seen success as a drug or payload delivery system in multiple mouse models and has even been used in clinical trials as a method to target IL-2 to metastatic melanoma (53, 62, 63). As a proof of concept study, Loeffler et al. designed an attenuated strain of *Salmonella typhirium* that expresses LIGHT and took advantage of the tumor-targeting characteristics to deliver the vector (53). BALB/c mice bearing 14-day D2F2 breast cancer tumors revealed significant reduction in tumor growth for mice that received Sal+LIGHT, an effect that was also observed in the metastasized D2F2 model through reductions in metastatic scores and lung tumor burden. Additionally, the authors were able to show that multiple treatments with i.v. Sal+LIGHT were effective 9-days post subcutaneous (s.c.) challenge in the CT-26 colon carcinoma model. The group then showed this therapeutic efficacy extended to other tumor models through significant reductions in tumor burden in C57BL/6 mice that had been challenged s.c. with LLC cells 7-days prior to the start of treatment. Mechanistic involvement of the LIGHT receptors HVEM and LTβR was indicated by including anti-LTβR and anti-HVEM antibodies in control groups that led to the loss of the anti-tumor effects of the vector (53).

Other methods that rely on engineered cells to target and deliver LIGHT to tumors have focused on the mesenchymal stem cell (MSC) population. Taking advantage of cancer endothelial cells' ability to attract MSCs (64, 65), Zou et al. developed a technique that utilizes MSCs expressing LIGHT, which resulted in LIGHT-expressing MSC trafficking to tumor sites (56). By inducing LIGHT expression in MSCs through lentiviral delivery of the vector *ex vivo*, Zou et al. utilized MSC-LIGHT in both a prophylactic (injection of MSC-LIGHT 13 days before challenge) and therapeutic manner (injection of MSC-LIGHT 7 days post challenge) in the TUBO mammary cancer model (56). Profound increases in the intra-tumoral CD4^+^ and CD8^+^ T cells were found in both treatment schedules as they repressed tumor growth compared to the controls. While tumors were unable to establish growth in the prophylactic setting, therapeutic intervention only controlled tumor growth (64). Interestingly, removing CD4^+^ T cells ablated MSC-LIGHT's prophylactic efficacy while removing CD8^+^ T cells removed MSC-LIGHT's therapeutic efficacy, suggesting different roles for each subset within this method of therapy (56). Anti-tumor memory was subsequently demonstrated through the inability of TUBO re-challenged mice to grow tumors. Importantly, this group established the role of LIGHT-LTβR signaling in tumor clearance by showing that an anti-LTβR antibody prevented therapeutic functioning of MSC-LIGHT, directly implicating LIGHT-LTβR interactions.

### Antibody and Peptide Fusion Proteins

Rather than using direct injection of virus, tumor homing cells, or bacteria, LIGHT has also been developed in recombinant peptide and fusion protein platforms that aim to combine the immunostimulatory effects of LIGHT with the ability to target tumor tissues. These moieties have used different strategies of fusing LIGHT to short tumor vasculature targeting peptide sequences (VTPs) or single-chain Fragment variable (scFv) antibodies that have historically been used as stand-alone treatments of cancer. In this manner, researchers can not only induce an anti-tumor immune response through LIGHT function, but also benefit from the targeting capabilities of VTP- or scFv-fused LIGHT moieties.

VTPs have been developed in such a manner that they preferentially interact with tumor angiogenic vessels, which are fundamentally different from healthy vasculature. VTP-fusion protein delivery has shown some limited success in clinical trials when the amino acid sequence CNGRCG (known as NGR) was fused to human TNFα. Specifically, when used in refractory solid tumors such as ovarian cancer in combination with doxorubicin, there was a measurable improvement in patient survival (66–72). Researchers sought to use this feature in an effort to deliver LIGHT systemically, thus eliminating the need for invasive delivery strategies such as intra-tumoral injection (32). Through the use of phage libraries, short peptide sequences were discovered that specifically target tumor angiogenic vasculature. Each VTP contains distinct tumor-specific vascular targets, potentially allowing functional delivery of LIGHT in multiple tumor types. As an example of the specificity that VTPs have in binding aberrant vasculature, the amino acid sequence CGKRK has been shown to preferentially bind tumor blood vessels as opposed to healthy vasculature, theoretically via heparan sulfates, phosphatidylserine, VEGF related extracellular matrices, or a combination of the three (69, 73).

Cancer models demonstrating the utility of LIGHT fused to CGKRK (LIGHT-CGKRK) include murine glioblastoma, murine pancreatic insulinoma, human astrocytoma and human grade I meningioma (22, 23, 74). Recently, the LIGHT-CGKRK fusion peptide was utilized to establish vascular normalization and improved perfusion in s.c. LLC and B16 melanoma models. Interestingly, the authors also showed that intravasation of LLC tumor cells into the bloodstream was decreased through early LIGHT- CGKRK interventions while establishment of visual lung metastatic events could be reduced with late LIGHT-CGKRK therapy that begins after surgical removal of primary tumors. They took this research even further by establishing that vascular normalization could occur within B16 lung metastases, and that LIGHT-CGKRK therapy was able to induce TLS formation at metastatic sites while also reducing metastatic burden (33). Another VTP with amino acid sequence CRGRRST (abbreviated RGR within the literature), binds specifically to platelet-derived growth factor receptor β (PDGFRβ), and is also successful in targeting LIGHT to murine pancreatic insulinoma and murine breast cancer. One additional benefit of the RGR peptide is that it also has the ability to bind to human glioblastoma tumor sections, which is an important finding for future translational efforts (21–23, 75, 76).

Additional approaches to engineering LIGHT-peptide proteins include fusing multiple monomers of LIGHT to tumor targeting antibodies. Tang et al. found success in this method by combining three units of modified LIGHT (hmLIGHT) that are able to bind and signal through both murine and human receptors with a functional chain (Fc) of immunoglobulin G (IgG) recognizing Epidermal Growth Factor Receptor (EGFR) (57). The product (anti-EGFR-hmLIGHT) was used to treat mice bearing Ag104Ld fibrosarcoma and MC38 colon adenocarcinoma (57). Anti-EGFR-hmLIGHT treatment induced complete tumor regression of small (7-days post s.c. injection) Ag104Ld-EGFR^+^ primary tumors as well as protected against re-challenge, but had little success as a monotherapy when the parental Ag104Ld tumor line was not over-expressing EGFR or tumors were older than 14 days (57). Tang et al. also reaffirmed that treatment was T cell dependent based on the 300 - 500% increase of intratumoral CD8^+^ T cells as well as increased IFNγ and TNFα levels. LIGHT-LTβR interaction was found to be the principle driver for this therapy due to the complete loss of anti-tumor effects when an anti-LTβR Ig was included.

## LIGHT Combination Therapies

Although some groups have shown that LIGHT can be used to a reasonable extent as a monotherapy, the most effective LIGHT-based interventions have come out of combinatory LIGHT-vector treatments together with either therapeutic vaccinations or checkpoint inhibitors.

### LIGHT + Therapeutic Vaccinations

Tumor vaccines are therapeutic vaccines that are given with the intent to stimulate an immune response directed against identified or neo-antigens occurring within tumors (77). Alone, they have not historically resulted in significant improvements to survival outcomes, however combining them with LIGHT has been shown to enhance effector cell function within tumors (42). To this end, multiple groups have demonstrated the benefits of combining therapeutic vaccines with LIGHT-based therapies (19, 22, 55).

Within the TRAMP-C2 prostate cancer tumor model, our group was able to show that the combination treatment of Ad-LIGHT with a prostate tumor associated antigen tumor vaccine (PSCA trivax) performed much better than Ad-LIGHT treatment alone (55). Mechanistically it was shown that Ad-LIGHT + PSCA trivax combination therapy increased intra-tumoral CD8^+^ T cells and prevented the maturation and functioning of intra-tumoral Tregs, ultimately creating a more immunologically hot tumor (55). Additional work from our group has illustrated the efficacy of Ad-LIGHT therapy in conjunction with anti-tumoral vaccines against human papillomavirus type (HPV)-transformed cancers (19). Within the HPV16 transformed tumor line, C3.43, the combination treatment of intra-tumoral Ad-LIGHT and HPV16-E7 expressing Venezuelan equine encephalitis virus replicon particles (VRP) as a tumor vaccine yielded significant regression of established tumors compared to Ad-LIGHT alone or HPV-VRP alone. This combination treatment lead to increased intra-tumoral anti-E7 CD8^+^ T cells as well as the presence of intra-tumoral inflammatory cytokines and activation markers IFNγ, IL-1a, MIG, and MIP-2. Furthermore, mice treated with Ad-LIGHT and the VRP vaccine were able to generate memory as 75% of mice remained tumor-free upon contralateral tumor re-challenge post-surgical resection of primary tumors (19).

### LIGHT + Checkpoint Inhibitors

Given that LIGHT-mediated changes to the TME facilitate the shift from a cold to a hot tumor phenotype, IFNγ levels also rise. Exposure to increased IFNγ mediates tumor upregulation of PD-L1 as a way to shut down immune responses (40). Taking advantage of this, researchers have found synergy with the combination treatment of LIGHT and anti-PD-L1 antibodies (78, 79). Tang et al. found that the combination treatment of anti-PD-L1 antibodies with anti-EGFR-hmLIGHT conferred the best treatment outcomes within their cancer models (57). As tumor size increased, LIGHT based therapy lost efficacy due to the tumor's elevated PD-L1 levels. Inhibiting PD-L1 allowed for further functioning of T cells via anti-EGFR-hmLIGHT within the Ag104Ld and MC38 tumor models, inducing complete rejection of established tumors in a therapeutic setting. Notably, monotherapy with either anti-EGFR-hmLIGHT construct or PD-L1 checkpoint inhibitor was ineffective at eliminating tumors (57, 80).

Combining LIGHT-VTP with anti-PD-1 and anti-CTLA-4 checkpoint inhibitors (dual checkpoint therapy) has also shown efficacy in combatting the tumor microenvironment (22). By utilizing LIGHT-VTP (CGKRK) and dual checkpoint therapy, Johansson-Percival et al. were able to confer a 6-week survival advantage along with vascular normalization and production of TLSs containing HEVs using the murine pancreatic insulinoma model (22). Furthermore, by including an anti-Tag-CpG-ODN tumor vaccine within Tag^+^ tumors, the triple treatment regimen elicited a 13-week survival improvement compared to LIGHT-VTP and dual checkpoint therapy (22). This was the first time LIGHT-VTP was utilized with both checkpoint inhibitors as well as a tumor vaccine. More recently, the effectiveness of LIGHT-VTP combined with an anti-PD-1 antibody was shown to dramatically improve long-term survival of mice bearing metastatic B16 lung tumors through significant reductions in quantifiable metastatic events. In line with the findings from primary tumor studies, the researchers found dramatic increases in HEV and TLS formation in metastatic tumors (33). Given the multiple promising outcomes from this work, further study of checkpoint inhibitor and tumor vaccine combination therapies are necessary for the future of LIGHT-based cancer therapies.

## LIGHT-Based Therapy Considerations and Future Directions

The autoimmune consequence of LIGHT overexpression is loss of peripheral tolerance, which has several implications for disorders such as inflammatory bowel disease, diabetes, asthma, graft vs. host disease, and even atherosclerosis (14, 81–87). Foundational studies have clearly shown that transgenic mice constitutively expressing LIGHT have a hyper-activated T cell population putting them at increased risk for spontaneous autoimmunity hallmarked by severe infiltration of effector cells within peripheral tissues. Because of this, it is of the utmost importance that measures be taken to ensure proper targeting of LIGHT vectors to desired sites and apply controlled dosages to prevent initiation of self-recognition. This may be especially important in future studies of LIGHT therapies if blood cancers are considered. One specific example of how this may be an issue is in the case of multiple myeloma (MM). Patients experiencing osteolytic lesions as a result of disease progression have shown significantly elevated levels of circulating LIGHT driven by activated CD8^+^ T cell, CD14^+^ monocytes, and neutrophils. When overproduced in MM patients, LIGHT synergizes with receptor activator of nuclear factor kappa-B ligand (RANKL) in driving osteoclast formation, resulting in a breakdown of long bones within the immediate areas of bone marrow (88). These recent results suggest that there are going to be certain cancers or individuals with autoimmune-related diseases that do not qualify for LIGHT-based immunotherapies as it may exacerbate disease manifestations.

Methods that will be most effective at minimizing harm from systemic LIGHT treatment will be enhanced targeted delivery to or controlled release of LIGHT treatments within target tissues. Forced expression of LIGHT by tumor tissues through the usage of viral vectors (e.g., Adenovirus) will almost certainly face issues of neutralizing immunity generated against the vector itself after the first treatment, therefore multiple serotypes or vectors will be required for this route of therapy to be effective. Other studies discussed within this review have shown the evolution of delivering LIGHT to the tumor from LIGHT-expressing bacterial cells to fusion protein constructs that have bimodal functions at tumor sites. These targeting strategies have shown great progress as they have the additional benefit of being combined, often successfully, with other immunotherapeutic interventions. It remains, however, that a significant factor needs to be considered in the application of LIGHT-based therapies in humans: decoy receptor 3 (DcR3). DcR3, also known as tumor necrosis factor receptor superfamily member 6b, is a functional attenuator of LIGHT signaling that is found in the genomes of humans but is absent in both mice and rats (89). While DcR3 serum levels are nearly undetectable in healthy individuals, those experiencing inflammatory disease and/or cancer see significant increases within the bloodstream. In the context of cancer, DcR3 has been found to be upregulated in astrocytoma and gliomas (90, 91). Furthermore, a positive correlation exists between expression of DcR3 and the severity of pancreatic carcinoma, colorectal cancer, breast, cervical, and ovarian cancers (89–93). These findings suggest that even if LIGHT therapy does move into the clinic, its effects may be dampened by a DcR3^+^ TME. As such, future methods that examine forced expression of DcR3 within mouse tumor models may serve to more appropriately represent a human TME and set up LIGHT-based therapies for a successful clinical transition, specifically informing whether a combination with an anti-DcR3 antibody would prevent attenuation of LIGHT functions.

LIGHT has not yet been used as a treatment in clinical trials. As such, translational studies that aim to move these constructs into humans will need to be considerate of the following: usage of human instead of murine LIGHT, validation of successful homo-trimerization of targeted LIGHT expression or recombinant LIGHT constructs, and verification of biological activity both *in vivo* and *in vitro* through the usage of anti-HVEM, anti-LTβR, soluble DcR3, or a combination of the three. The construct created by Tang et al. has made significant strides in these areas as they linked three repeats of a reengineered form of LIGHT that has an affinity for and shows functionality with both mouse and human receptors, a feature lacking in other LIGHT-based designs. Additionally, they were able to show that their fusion protein had direct effects on the activation of relevant immune cell populations *in vitro*. These controls have been lacking in other peptide-based delivery vectors and should not be overlooked. It is the opinion of our group that this design is superior to its predecessors and is more likely to produce a functional LIGHT construct that will function in both mouse and human studies.

Future approaches such as engineered exosomes containing LIGHT decorated with tumor-targeting moieties may provide a method of shielding LIGHT protein from degradation within the blood stream while allowing transport to tumor sites for delivery. This method may effectively deliver LIGHT payloads to tumor sites. However, there may be significant hurdles in maintaining surface expression on target cells as it is not known to what degree exosome endocytosis will occur in different tumor models. Additionally, in combination with next generation chimeric antigen receptor (CAR) T cells, delivery of LIGHT to the TME may finally provide a breakthrough in CAR-T infiltration and activity in solid tumors. Either an effective delivery system combined with CAR-T therapy or generation of an armored CAR-T cell that produces a LIGHT-related construct once engaged with its target should be investigated. Strategies such as this will also see benefits from the generation of neo-antigen responses by the patient's immune system as LIGHT stimulates NK cell activity, DC antigen presentation, and T cell expansion (12). Given the efficacy of CAR-T therapies for blood-based cancers it may be required to include a negative feedback switch, such as a tyrosine-kinase inhibitor (ex. dasatanib), alongside treatment to control responses or the usage of lower-affinity TCRs (94, 95).

## Conclusions

Despite improvements in immunotherapy, eliciting a robust anti-tumor immune response with the ability to infiltrate clear established tumors remains a challenge. LIGHT-based therapies have shown great effectiveness in reducing tumor burden and generating lasting anti-tumor memory by modifying the TME through normalizing tumor vasculature, driving TLS neo-genesis at tumor sites that contain HEV, and dramatically improving effector TIL infiltration. The insights that LIGHT research has provided in the recent decades warrants continued investigation of its use as a cancer therapeutic, especially since the effects of LIGHT-supported immunotherapy combinations can be seen in both the primary and metastatic settings of multiple tumor types when the vector for delivery functions as intended.

## Author Contributions

JS conceptualized, outlined, researched, wrote, generated the figure & table, and handled the review/editing process of the manuscript. MO, RP, DF, DD, and WK provided research, input on figure & table, and editing of the manuscript.

## Conflict of Interest

The authors declare that the research was conducted in the absence of any commercial or financial relationships that could be construed as a potential conflict of interest.
